# Chlorin e6-Induced Photodynamic Effect Polarizes the Macrophage Into an M1 Phenotype Through Oxidative DNA Damage and Activation of STING

**DOI:** 10.3389/fphar.2022.837784

**Published:** 2022-03-03

**Authors:** Ting-Ting Yu, Ning Han, Liu-Gen Li, Xing-Chun Peng, Qi-Rui Li, Hua-Zhen Xu, Xi-Yong Wang, Zi-Yi Yang, Xiao Chen, Mei-Fang Wang, Tong-Fei Li

**Affiliations:** ^1^ School of Basic Medical Sciences, Hubei University of Medicine, Shiyan, China; ^2^ Department of Respiratory, Taihe Hospital of Shiyan, Hubei University of Medicine, Shiyan, China; ^3^ Hubei Key Laboratory of Embryonic Stem Cell Research, Hubei University of Medicine, Shiyan, China; ^4^ Department of Pharmacology, School of Basic Medical Sciences, Wuhan University, Wuhan, China

**Keywords:** macrophages, photodynamic effect, reactive oxygen species (ROS), DNA damage response (DDR), STING molecule, autophagy

## Abstract

The tumor-associated macrophage (TAM) serves as an immunosuppressive agent in the malignant tumor microenvironment, facilitating the development and metastasis of lung cancer. The photodynamic effect destabilizes cellular homeostasis owing to the generation of reactive oxygen species (ROS), resulting in the enhanced pro-inflammatory function of immunocytes. In our previous study, the Ce6-mediated photodynamic effect was found to have kept the viability of macrophages and to remodel them into the M1 phenotype. However, the mechanism remains unrevealed. The present study now explores the mechanism of photodynamic therapy (PDT)-mediated reprogramming of macrophages. As expected, Ce6-mediated PDT was capable of generating reactive oxygen species, which was continuously degraded, causing “low intensity” damage to DNA and thereby triggering subsequent DNA damage response in macrophages. The autophagy was thus observed in Ce6-treated macrophages and was shown to protect cells from being photodynamically apoptotic. More importantly, Ce6 PDT could activate the stimulator of interferon genes (STING) molecule, a sensor of DNA damage, which could activate the downstream nuclear factor kappa-B (NF-κB) upon activation, mediating the polarization of macrophages towards the M1 phenotype thereupon. In addition, inhibition of ROS induced by PDT attenuated the DNA damage, STING activation, and M1-phenotype reprogramming. Furthermore, the silence of the STING weakened Ce6 treatment-mediated M1 remodeling of macrophages as well. Altogether, these findings indicate the Ce6-induced photodynamic effect polarizes macrophages into an M1 phenotype through oxidative DNA damage and subsequent activation of the STING. This work reveals the crucial mechanism by which photodynamic therapy regulates the macrophage phenotype and also provides a novel intervenable signaling target for remodeling macrophages into the M1 phenotype.

**Graphic Abstract:**
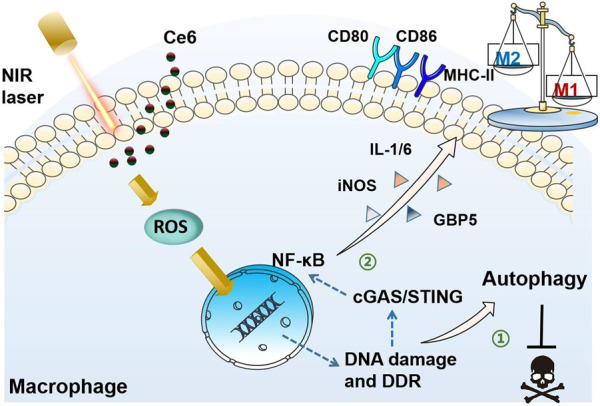


## Introduction

Macrophages, especially tumor-associated macrophage (TAM), are crucial immunocytes in the lung cancer microenvironment, accounting for a notable proportion, up to 50% in a variety of solid tumors (Li et al., 2018; Singhal et al., 2019; Larionova et al., 2020; Sedighzadeh et al., 2021). Their actions in promoting immunosuppression in lung cancer by releasing immunosuppressive cytokines, reduction of cytotoxic lymphocyte infiltration, and increasing the ratio of regulatory T cells (Treg) contribute to the failure of eradicating lung cancer (Economopoulou and Mountzios, 2018; Xu et al., 2020; Sarode et al., 2020; La Fleur et al., 2021). However, it is worth noting that macrophages are phenotypically remodeled and can be re-polarized into the immunostimulatory M1 phenotype under certain conditions to perform antitumor effects, which provides an important intervention target for anti–lung cancer immunotherapy (Li et al., 2017; Li et al., 2019; Wang et al., 2019). We have previously found that photodynamic effects mediated by a certain condition of chlorin e6 (Ce6) treatment could reprogram macrophages to the M1 phenotype and thereby act as an anti–lung cancer immunotherapeutic agent (Yu et al., 2021). However, the mechanism of macrophage reprogramming by Ce6 treatment is not yet clear.

Photodynamic therapy (PDT) has been reported to generate reactive oxygen species (ROS) that produce oxidative damage to organelles, especially capable of damaging DNA in the nucleus (Cadet and Wagner, 2013; Xie et al., 2018; Srinivas et al., 2019; Cal et al., 2020). The stimulator of interferon genes (STING) is the recently discovered essential molecule that mediates inflammatory responses and immune responses. The activation of STING molecules by viral DNA and DNA fragments of tumor cells induces anti-infective and antitumor immune responses (Chen et al., 2016; Li et al., 2018; Motwani et al., 2019; Kwon and Bakhoum, 2020). Ce6-mediated photodynamic action should activate the intracellular STING after damaging the DNA of lung cancer cells *via* ROS, which resulted in the activation of the downstream nuclear factor kappa-B (NF-κB) (Hou et al., 2018; Dunphy et al., 2018; Sun et al., 2020). NF-κB, an important nuclear transcription factor, promotes the expression of inducible nitric oxide synthase (iNOS), guanylate binding protein 5 (GBP5), major histocompatibility complex-II (MHC-II), CD80, and CD86, which are biomarkers of the M1 macrophage, polarizing macrophages into the antitumor M1 phenotype. On the other hand, ROS-induced DNA damage is also known to activate the DNA damage response (DDR), which can lead to elevated levels of autophagy (Galati et al., 2019; Roshani-Asl et al., 2020; Wang et al., 2021). This is possibly the reason why, under a certain condition, Ce6-mediated PDT does not destroy macrophages.

Based on the aforementioned evidence, we speculated that the Ce6-induced photodynamic effect polarizes the macrophage into the M1 phenotype through oxidative DNA damage and subsequent activation of the STING. Herein, the macrophages were remodeled into the M1 phenotype characterized by the enhanced expression of CD80, CD86, MHC-II, iNOS, GBP5, IL-1β, and IL-6 under the treatment of Ce6 PDT. Further, Ce6 PDT was found to facilitate ROS generation and DNA damage without inducing apoptosis of macrophages. The enhanced autophagy also could be observed in Ce6-treated macrophages. Inhibition of autophagy led to the loss of viability and increased apoptosis in macrophages treated with Ce6. Additionally, molecules of the STING and NF-κB were activated by Ce6 PDT. Notably, inhibition of ROS induced by Ce6 PDT attenuated the DNA damage, STING activation, and M1-phenotype reprogramming. Finally, the silence of the STING weakened Ce6 treatment-mediated M1 remodeling of macrophages.

## Materials and Methods

### Cell Cultures

RAW 264.7 cells (Macrophages, ATCC number: TIB-71), a typical mouse macrophage cell line, were utilized as the macrophage model (Zhou et al., 2018; Zumerle et al., 2019; Wu et al., 2019). Lewis lung carcinoma (LLC, ATCC number: CRL-1642), a mouse lung carcinoma cell line, was performed as a lung cancer cell model (Zhao et al., 2019). These two cell lines were purchased from the Cell Bank of Shanghai Institutes for Biological Sciences (Shanghai, China). All cells were cultured in the RPMI-1640 medium or DMEM (Sigma-Aldrich, St Louis, United States) supplemented with 10% fetal bovine serum (Sigma-Aldrich, St Louis, United States) in an incubator with humidification (5% CO_2_ and 95% air ambience, 37°C). The RPMI-1640 medium was applied to culture RAW 264.7 cells, while the DMEM was used to develop Lewis cells. When the two types of cells were co-cultured, the RPMI-1640 medium was adopted.

### Cell Treatments

Ce6, used as a photodynamic agent in the present study, was purchased from Macklin (19660-77-7, Macklin-Lab, Shanghai, China). Macrophages were incubated with Ce6 (4 μg/ml) for 12 h and then irradiated with laser (the wavelength was 690 nm, and the power of the laser was 200 mW) for 20, 40, or 60 s (In some experiments, just one of these conditions was selected). The cells were then left for 8 h.

### Phenotype and Functional Assay of Macrophages

Proteins of macrophages were harvested. The expression of iNOS and GBP5 was assayed by Western blotting (WB). Alternatively, cells were harvested and washed with PBS three times; the expression of surface MHC-II was detected by immunofluorescence staining and analyzed by flow cytometry (CytoFLEX, Beckman Coulter, United States). In addition, mRNA levels of IL-6 and IL-1β were detected by RT-PCR (CFX96 Touch, Bio-Rad). Phagocytosis of macrophages was analyzed by fluorescent latex beats and confocal laser scanning microscopy (confocal microscopy, FV3000RS, Olympus) (Akilbekova et al., 2015). Briefly, the activated latex beats were incubated with Ce6-treated macrophages for 1–2 h. Cells were then washed with PBS three times and fixed using 4% paraformaldehyde, stained with Hoechst 33342, and then observed by confocal microscopy. Alternatively, Ce6-treated macrophages were co-cultured with LLC for another 24 h. As macrophages have stronger adhesion ability than LLC, the time difference can be utilized to harvest LLC first for apoptosis detection. Annexin-V/PI double staining and flow cytometry were employed to detect the apoptosis rate of cells.

### ROS Detection and Inhibition

Macrophages were seeded in 24-well plates with a density of 3 × 10^5^ cells per well and treated as mentioned earlier and then left for 2, 4, 6, and 8 h, respectively. The cells were then incubated with 10 μM of 2,7-Dichlorodi-hydrofluorescein diacetate (DCFH-DA, S0033, Beyotime, Shanghai, China) at 37°C for 30 min before being harvested and assayed by flow cytometry. Alternatively, N-Acetyl-l-cysteine (NAC, 3 mM) was used to pre-treat macrophages for 2 h to block the generation of ROS.

### DNA Damage and Subsequent Response Analysis

DNA double-strand break (DDSB) was detected by the comet assay. Briefly, macrophages were seeded in 24-well plates and treated with Ce6. Cells in PBS were prepared and mixed with low melting point agarose. The mixture was then dripped onto a glass slide pre-coated with agarose gel and pressed, followed by electrophoresis at 25 V, 300 mA for 25 min. The mixture was then neutralized using tris-Hcl (PH = 6.0) (Olive and Banáth, 2006). Finally, the nuclear dye was used to stain nuclei. Samples were photographed by fluorescence microscopy (IX53+DP73, Olympus). For DDR analysis, cells in the 6-well plates were treated with Ce6. Total proteins were extracted for the WB assay of γ-H2A.X, Bax, caspase-3, and p53 expressions.

### Autophagy Detection and Inhibition

Macrophages were seeded in a 6-well plate or on a confocal dish with the treatment of Ce6. The proteins wherein cells were harvested and used for WB to verify the expression of LC3-II/LC3-I, Atg5, and Beclin-1. The cells on the confocal dish were fixed with 4% paraformaldehyde and incubated with the LC-3 antibody and fluorescent secondary antibody and then imaged by laser scanning confocal microscopy (FV3000RS, Olympus, Japan). For inhibition of autophagy, wortmannin (300 nM) was applied to incubate with macrophages for 1 h and then removed. The cells were treated with Ce6, as described earlier. The autophagy-related proteins were detected. Cell apoptosis and apoptosis-associated proteins were then assayed.

### Macrophage Viability Assay

Macrophages were plated in 96-well plates with a density of 6 × 10^3^ cells per well and treated, as mentioned earlier. The cell viability was detected using a CCK-8 kit (HY-K0301, MCE, NJ, United States). On the other hand, proliferation and apoptosis of macrophages were measured by the Annexin-V/PI staining assay and WB analysis of Bax and caspase-3.

### Assay of Activation of the STING and NF-κB

Macrophages were seeded in 6-well plates or on a confocal dish and treated, as mentioned earlier. First, the expression of NF-κB, p-NF-κB, STING, and cGAS was detected by WB. Alternatively, the nuclear translocation of NF-κB was analyzed by immunofluorescence staining of NF-κB and confocal microscopy (FV3000RS, Olympus, Japan).

### Silence of the STING

The STING expression in macrophages was silenced using small interference RNA (SiRNA). The SiRNA nucleotides of targeting mouse STING (5′-GGA​UCC​GAA​UGU​UCA​AUC​Att-3′) were purchased from GenePharma (Suite 602, 1011 Halley Road, Zhangjiang Hi-Tech Park, Shanghai). SiRNAs were transfected into macrophages in the presence of the lipofectamine 3000 reagent, according to the manufacturer’s recommended procedure. Briefly, 5 μl of lipofectamine 3000 and 100 pM of SiRNA were used to transfect macrophages after co-incubation in 250 serum-free media for 15 min. After 8 h, the SiRNA and transfection reagent were withdrawn. An efficient knockdown of the STING was assayed by WB. The non-silencing SiRNAs (Scramble) were used as a control. After transfection, subsequent Ce6 PDT was processed on macrophages.

### Annexin-V/PI Assay of Apoptosis

For the assay of apoptosis, macrophages or LLC was harvested and then washed with PBS three times. Cells were incubated with FITC-Annexin-V (Purchased from CHAMOT BIOTECHNOLOGY CO., LTD.) for 10 min and then incubated with PI for another 5 min. Cells were monostained with FITC-Annexin-V or PI as a control for compensation. The cellular fluorescence of Annexin-V and PI was detected by flow cytometry (CytoFLEX, Beckman Coulter, United States).

### Immunofluorescence Staining of NF-κB, LC-3, CD80, CD86, and MHC-II

For the detection of NF-κB, LC-3, and MHC-II expressions or location, cells were incubated with primary antibodies of NF-κB (10745-1-AP, Proteintech, Wuhan, China), LC-3 (12741S, CST, Boston, United States), CD80 (E-AB-F0992D, Elabscience), CD86 (E-AB-F0994D, Elabscience), and MHC-II (sc-66205, Santa Cruz Biotechnology, Santa Cruz, United States) overnight at 4°C and then incubated with the goat anti-rat IgG/Alexa Fluor 488 secondary antibody (bs-0293G-AF488, Bioss, Beijing, China) for another 60 min and washed three times before confocal microscopy (FV3000RS, Olympus, Japan) or flow cytometry (CytoFLEX, Beckman Coulter, United States). CD80 and CD86 were direct-labeled antibodies, and no secondary antibody incubation was required.

### Flow Cytometry Analysis

Ce6 fluorescence was acquired in the APC channel. CFSE fluorescence or Alexa Fluor 488 secondary antibody fluorescence was acquired in the FITC channel. PI fluorescence was acquired in the PE channel. The excitation wavelength was 638 nm, and the emission wavelength was 660 nm in the APC channel, 488 and 525 nm in the FITC channel, 561 and 585 nm in the PE channel, respectively. After cells were processed and collected as described earlier, they were filtered into special tubes for flow cytometry. Each channel was adjusted to the appropriate voltage before collecting cells. At least 1 × 10^4^ cells per sample were acquired for every collection. Geometric means (GM) were used to quantify the mean fluorescent intensity (MFI).

### Western Blotting(WB) Assay

Cells treated as described earlier were washed three times with PBS and lysed in RIPA buffer with a 1% protease inhibitor. Cell lysates were centrifuged, and the protein concentration was measured using a BCA assay kit. Equal protein aliquots (10 μg) were fractionated by SDS-PAGE and transferred to a PVDF membrane. The membranes were blocked with 3% bovine serum albumin in TBST and incubated with primary antibodies of Bax (bs-0127R, Bioss, Beijing, China), p53 (bs-2090R, Bioss, Beijing, China), γ-H2A.X (bs-3185R, Bioss, Beijing, China), PCNA (bs-2006R, Bioss, Beijing, China), LC-3 (12741S, CST, Boston, United States), Atg-5 (10181-2-AP, Proteintech, Wuhan, China), Beclin-1 (bs-1353R, Bioss, Beijing, China), NF-κB (10745-1-AP, Proteintech, Wuhan, China), p-NF-κB (bs-0982R, Bioss, Beijing, China), STING (19851-1-AP, Proteintech, Wuhan, China), cGAS (ab252416, Abcam, Cambridge, United Kingdom), iNOS (ab15323, Abcam, Cambridge, United Kingdom), GBP5 (13220-1-AP, Proteintech, Wuhan, China), Caspase-3 (50599-2-Ig, Proteintech, Wuhan, China), and GAPDH (PMK053C, BioPM, Wuhan, China) overnight at 4°C, and then, the membranes were incubated with the horseradish peroxidase-conjugated secondary antibody. Finally, protein bands were developed using an ECL, and the films were exposed using a bio-imaging system (170-8265, Bio-Rad).

### RT-PCR Assay

mRNAs of cells were extracted and reverse transcribed to cDNA. cDNA was amplified using an SYBR Green qPCR Master Mix kit (PC3301, Beijing, Aidlab). RT-PCR was performed using a Bio-Rad CFX Connect optics module, and data were analyzed using Bio-Rad CFXmanager. The specific primer sequences are as follows:

Mouse IL-1β Forward: GCA​ACT​GTT​CCT​GAA​CTC​AAC​T.

Mouse IL-1β Reverse: ATC​TTT​TGG​GGT​CCG​TCA​ACT.

Mouse IL-6 Forward: CGG​AGA​GGA​GAC​TTC​ACA​GAG.

Mouse IL-6 Reverse: ATT​TCC​ACG​ATT​TCC​CAG​AG.

Mouse GAPDH Forward: AGG​TCG​GTG​TGA​ACG​GAT​TTG.

Mouse GAPDH Reverse: TGT​AGA​CCA​TGT​AGT​TGA​GGT​CA.

### Mouse Lung Cancer Model and Histology Analysis

Female C57 mice at 4–6 weeks (20–22 g) were purchased from the Laboratory Animal Center at the Hubei University of Medicine (Hubei, China). Animal handling and experimental procedures were in line with protocols approved by the Animal Care Committee at the Hubei University of Medicine. Mice were housed in a temperature-controlled environment with fresh water and rodent diet available at all times. All inoculations and administrations were performed under nembutal anesthesia. To determine the role of Ce6 PDT in the reprogramming of macrophages, DNA damage, levels of autophagy, and activation of the STING, NF-κB, subcutaneous lung cancer cell (2 × 10^6^ cells/150 μl in PBS)-bearing mice received irradiation (690 nm, 200 mW, 40 s) after Ce6 administration (1 mg/kg bw). For about 12 h, the mice were sacrificed, and tumor grafts were harvested. Paraffin sections of tumor tissues were dewaxed, rehydrated, and antigen repaired with sodium citrate for 20 min. The paraffin sections were then incubated in 3% hydrogen peroxide for 12 min at room temperature. The paraffin sections were blocked with 5% BSA for 40 min, stained with primary antibodies overnight at 4°C, and then stained with the secondary antibody (PV-9000, ZSGB-BIO, Beijing, China) for 1 h at 37°C. Diaminobenzidine (DAB, ZLI-9018, ZSGB-BIO, Beijing, China) was applied for coloration for 1–3 min at room temperature. Hematoxylin was used to stain the nucleus. Primary antibodies included CD11b (20991-1-AP, Proteintech, Wuhan, China), iNOS (ab15323, Abcam, Cambridge, United Kingdom), GBP5 (13220-1-AP, Proteintech, Wuhan, China), MHC-II (sc-66205, Santa Cruz Biotechnology, Santa Cruz, America), γ-H2A.X (bs-3185R, Bioss, Beijing, China), p53 (bs-2090R, Bioss, Beijing, China), cGAS (ab252416, Abcam, Cambridge, United Kingdom), STING (19851-1-AP, Proteintech, Wuhan, China), NF-κB (10745-1-AP, Proteintech, Wuhan, China), LC-3 (12741S, CST, Boston, United States), Atg-5 (10181-2-AP, Proteintech, Wuhan, China), and Beclin-1 (bs-1353R, Bioss, Beijing, China). Finally, the paraffin sections were observed by orthomorphic Olympus microscopy (BX53+DP74, Olympus).

### Statistical Analysis

All statistics were presented using the mean ± standard deviation (SD). Statistical differences between groups were analyzed using one-way analysis of variance (ANOVA). *p* values < 0.05 were considered to be statistically significant.

## Results

### Ce6-Mediated Photodynamic Effect Polarized Macrophages Into the M1 Phenotype

**FIGURE 1:**
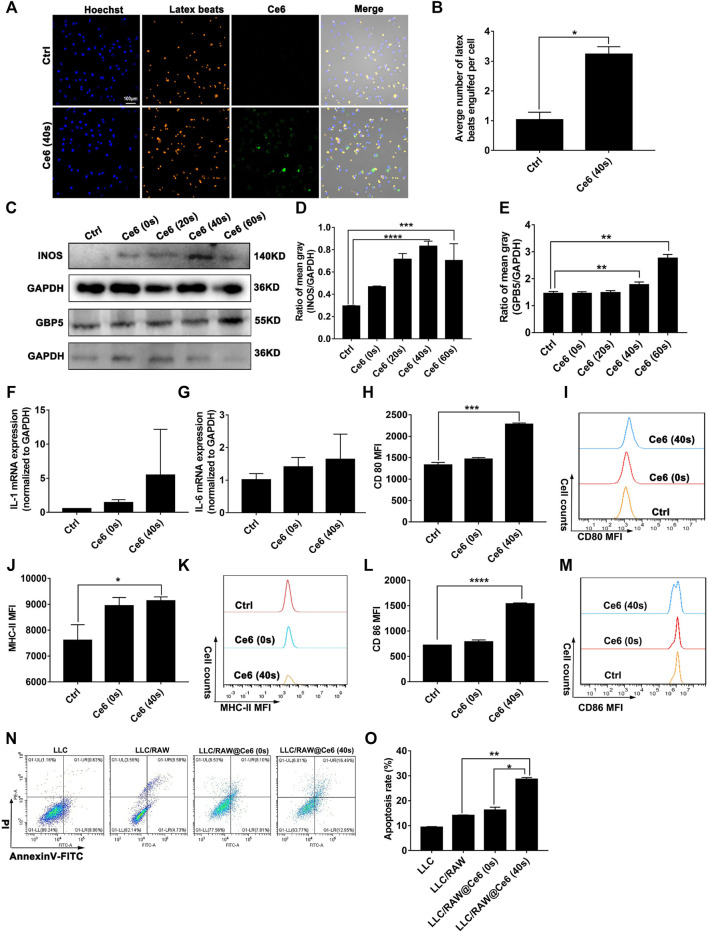
Ce6 PDT remodeled macrophages into the M1 phenotype *in vitro*. Macrophages were treated by Ce6 PDT (Ce6 was loaded at a concentration of 4 μg/ml, the loading time was 12 h, the irradiation time was 20/40/60 s, and the placement time was 8 h). **(A,B)** Phagocytic ability of macrophages was assayed through the fluorescence latex beats experiment and confocal microscopy. The average number of latex beats engulfed by macrophages was counted. **(C–E)** Expressions of GBP5 and iNOS were measured by WB. The blots were quantitatively analyzed using the ratio of mean gray. **(F,G)** mRNA levels of IL-1β and IL-6 were quantified through RT-PCR. **(H–M)** Surface CD80, CD86, and MHC-II expressions were detected by immunofluorescence staining and flow cytometry. Geometric means were used to quantify the MFI. **(N,O)** LLC were co-cultured with macrophages treated with Ce6, as described before. The apoptosis rate of LLC was detected by Annexin-V/PI double staining and flow cytometry. Values were means ± SD (*n* = 3, *p < 0.05, **p < 0.01, ***p < 0.001, ****p < 0.0001).

**FIGURE 2:**
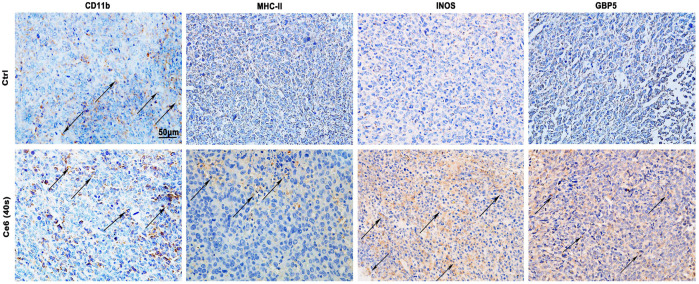
Ce6 PDT promoted upregulation of MHC-II, iNOS, and GBP5 in tumor grafts of LLC-bearing mice. Subcutaneous lung cancer cell-bearing mice received irradiation (690 nm, 200 mW, 40 s) after Ce6 PDT. Tumor grafts were harvested after 12 h. The biomarker of macrophages (CD11b) was detected by the IHC assay. In addition, MHC-II, iNOS, and GBP5 molecules, which are biomarkers of type-I macrophages, were analyzed by IHC.

**FIGURE 3:**
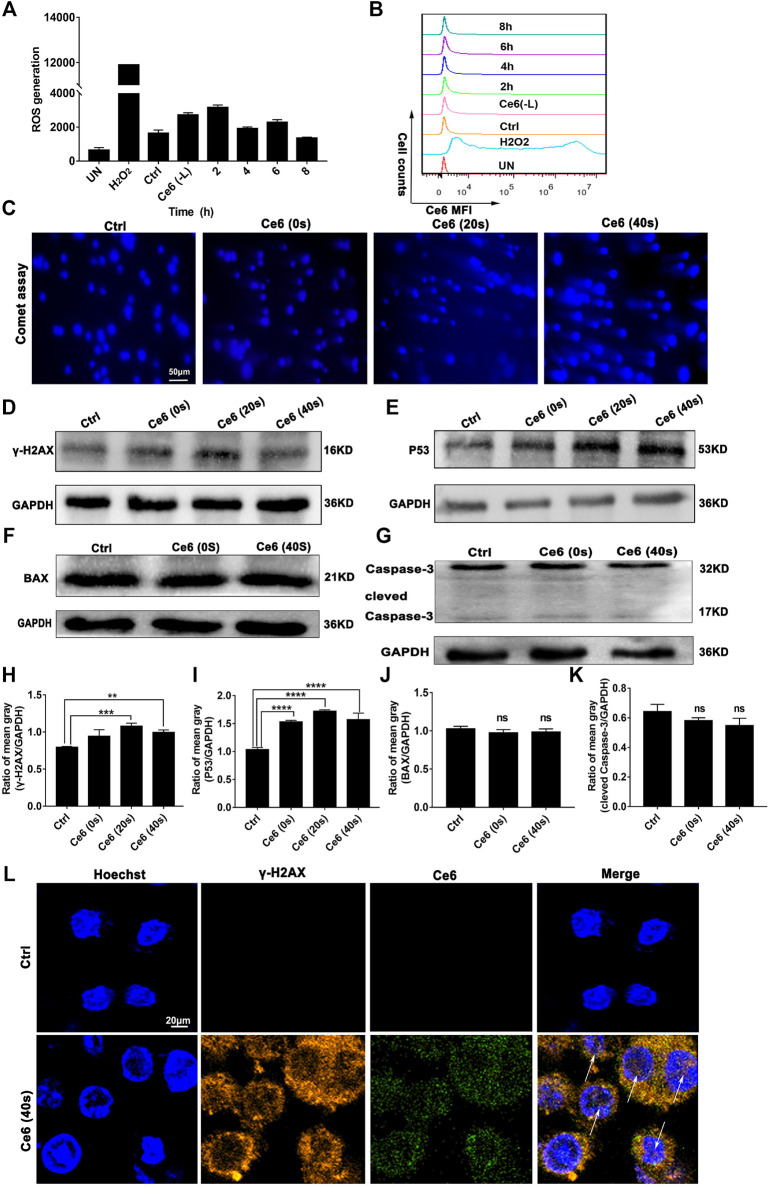
PDT induced by Ce6 promoted ROS production and DNA damage *in vitro*. **(A,B)** Intracellular ROS generation was detected by the DCFH-DA probe and flow cytometry. Geometric means were used to quantify the MFI. **(C)** The DNA breakage in macrophages was assayed using the comet assay. **(D,E)** The activity of DDR characterized by the enhanced expression of p53 and γ-H2A.X was detected by WB. **(F,G)** The expression of apoptotic proteins Bax and caspase-3 was measured by WB. **(H–K)** The blots were quantitatively analyzed using the ratio of mean gray. **(L)** The cellular distribution and expression of γ-H2A.X were analyzed by immunofluorescence staining. Values were means ± SD (*n* = 3, *p < 0.05, **p < 0.01, ***p < 0.001, ****p < 0.0001).

In our previous study, macrophages were shown to deliver the photosensitizer Ce6 to lung cancer cells and were reprogrammed to the M1 phenotype by the photodynamic effect, thereby inhibiting lung cancer (Yu et al., 2021). According to these, in the present research study, Ce6 (4 μg/ml) was loaded for 12 h (irradiation time was 40 s, and the laser power was 200 mW). The cells were left for 8 h after Ce6 treatment. Indeed, as shown in Figures 1A,B, the phagocytic function of macrophages was enhanced after the photodynamic action. More meaningfully, the co-culture model of macrophages and LLC demonstrated that the photodynamic action induced by Ce6 promoted the inhibitory effect of LLC by macrophages, characterized by an elevated apoptosis rate of LLC (Figures 1N,O). Additionally, the expressions of iNOS, GBP5, surface MHC-II, CD80, CD86, IL-1β, and IL-6 (Figures 1C–M; Supplementary Figure S2), which are characteristics for type-I macrophage activation, were upregulated in Ce6-treated macrophages. Consistently, *in vivo* experiments showed significant upregulation of iNOS, GBP5, and MHC-II in tumor grafts of LLC-bearing mice that received Ce6 PDT, suggesting that more M1-phenotype macrophages may be present in tumor tissues (Figure 2). The expression of CD11b in tumor grafts indicated macrophage infiltration in the lung cancer tissue, which was a prerequisite for photodynamic remodeling of macrophage *in vivo* experiments (Figure 2). Notably, as shown in Supplementary FigureS1, we explored the effect of laser light duration on the macrophage viability and found that cell viability decreased at 60 s of light duration. Based on it, we speculated that the inhibition of cell viability brought about by the excessive photodynamic effect may be one of the reasons affecting their intracellular expression of various proteins. Consequently, the upregulation of iNOS and γ-H2A.X expression (Figures 1C, 3D) induced by the excessive photodynamic effect may be inhibited. In a word, all evidence demonstrated the photodynamic effect induced by Ce6-reprogrammed macrophages into the M1 phenotype and augmented its suppressive function on lung cancer cells.

### Ce6 PDT Facilitated ROS Generation and DNA Damage Response

**FIGURE 4:**
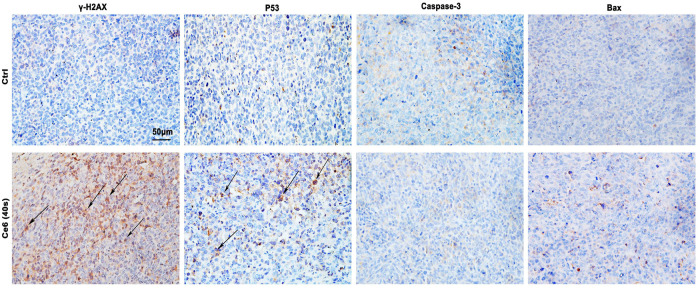
PDT induced by Ce6 led to DNA damage *in vivo*. p53 and γ-H2A.X expressions, which indicated the activation of DDR, were analyzed by IHC staining. Moreover, the expression of Bax and caspase-3 was also detected by IHC assay.

To verify whether Ce6 PDT was able to induce efficient DNA damage and DNA damage response (DDR), the levels of ROS generated by Ce6 PDT and its degradation over time were thus investigated initially. As shown in Figures 3A,B, intracellular ROS production was somewhat elevated just after irradiation and degraded after 8 h. Such a low ROS could cause “low intensity” DNA damage and subsequent repair response rather than leading to cell apoptosis. We thereof proceeded to examine the DDR of lung cancer cells after Ce6 PDT. In consequence, DDR, as indicated by the enhanced expression of p53 and γ-H2A.X, was prominent in macrophages that received Ce6 PDT (Figures 3D,E,H,I,L). Interestingly, according to references [32, 33], p53 has been shown to be involved in macrophage polarization, (Li et al., 2015) which corroborated with the results of macrophage phenotypic M1 remodeling in Figures 1, 2. In addition, the comet assay directly proved the DNA double-strand breakage (DDSB) in Ce6-treated macrophages (Figure 3C). DNA damage and DDR, on the one hand, can induce apoptosis when the damage of DNA is strong. Therefore, to verify the apoptosis of macrophages, downstream molecules of p53, such as Bax and caspase-3 were investigated. Notably, macrophages exhibited almost invariable apoptosis under PDT conditions used in the present study (Figures 3F,G,J,K). We speculated that the milder PDT conditions we used caused lower ROS and thus caused “low intensity” DNA damage, which was not sufficient to directly induce apoptosis of macrophages. In corroboration to the *in vitro* observations, p53 and γ-H2A.X-positive cells were increased in tumor tissues of mice treated with Ce6 (Figure 4), indicating that cells (both macrophages and tumor cells) in the tumor tissues underwent DNA damage, whereas the expression of Bax and caspase-3 varied little (Figure 4). A minor proportion of positive cells may be derived from apoptotic tumor cells that may have been attacked by reprogrammed M1-phenotype macrophages. Taken together, the Ce6 PDT under certain conditions resulted in a generation of a low level of ROS, thus promoting “low intensity” DNA damage and DDR in macrophages.

### Autophagy Triggered by Ce6 PDT Protected Macrophages From Apoptosis

**FIGURE 5:**
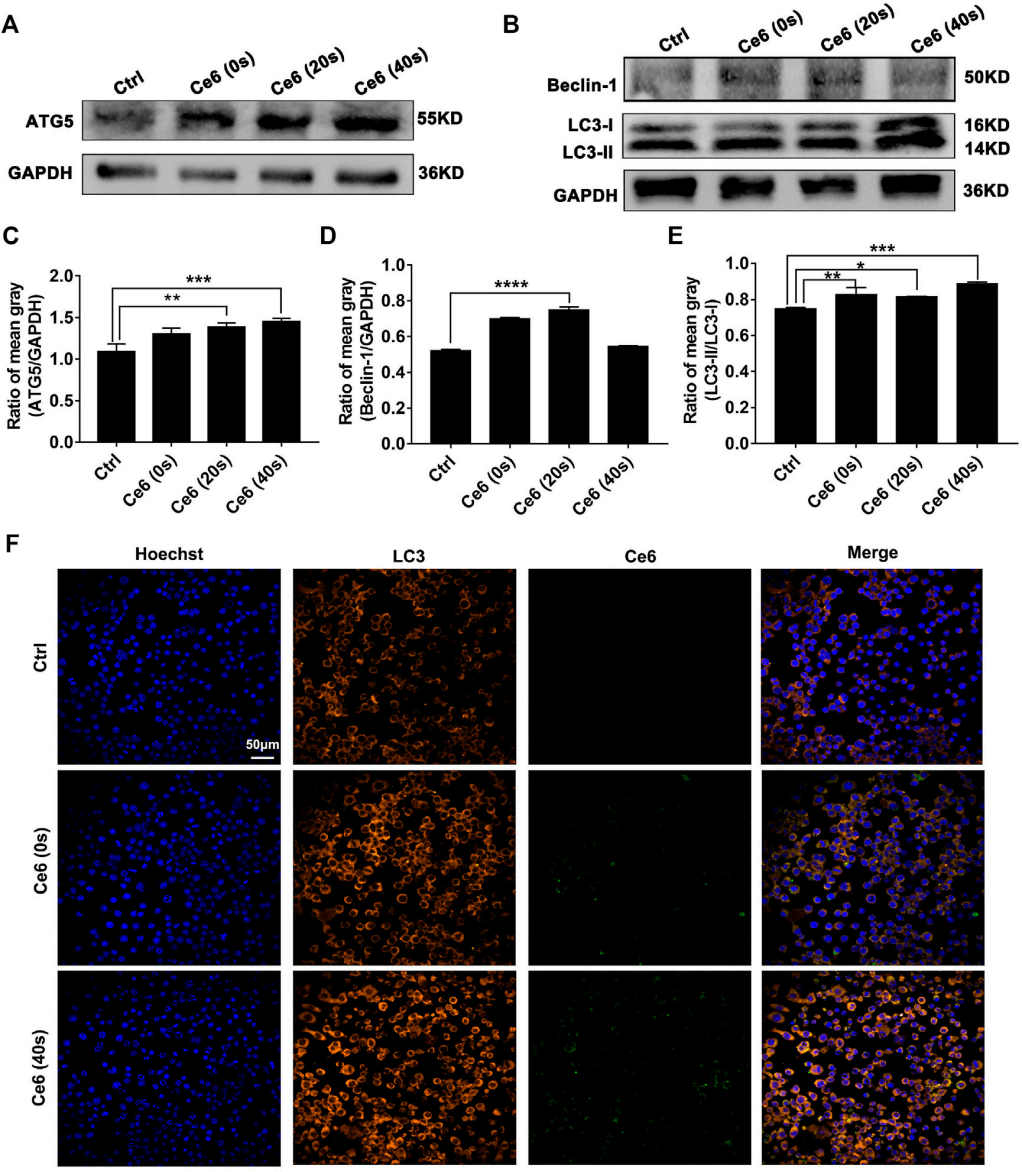
Photodynamic effect facilitated autophagy of macrophages *in vitro*. **(A–E)** Expressions of Atg-5, Beclin-1, and LC-3 II were assayed by WB. The blots were quantitatively analyzed using the ratio of mean gray. **(F)** The expression and distribution of LC-3 were analyzed by immunofluorescence staining. Values were means ± SD (*n* = 3, *p < 0.05, **p < 0.01, ***p < 0.001, ****p < 0.0001).

**FIGURE 6:**
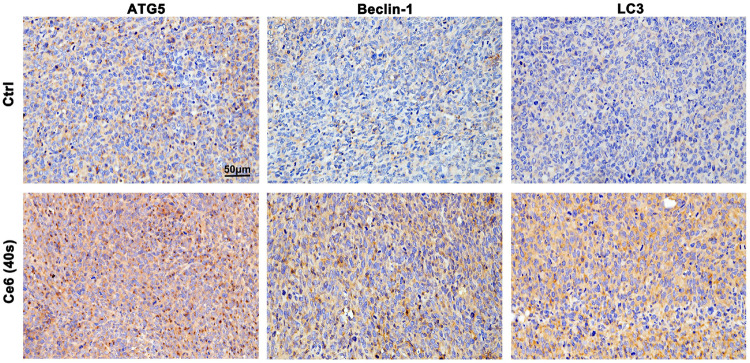
Ce6 PDT triggered autophagy in tumor grafts of LLC-bearing mice. IHC staining indicated pronounced upregulation of Atg-5, Beclin-1, and LC-3 II in tumor grafts that received Ce6 treatment.

In addition to inducing apoptosis, the DNA damage response (DDR) on the other hand, has been reported to activate autophagy (Galati et al., 2019; Roshani-Asl et al., 2020; Wang et al., 2021), which may protect macrophages from death thereupon. Following this, we evaluated the level of autophagy in macrophages that received Ce6 PDT thereupon. As shown in Figures 5A–E, the pronounced autophagy, as indicated by the enhanced expression of Atg-5, Beclin-1, and LC-3 II, appeared in Ce6-treated macrophages. Meanwhile, immunofluorescence staining also confirmed the enhanced expression and intracellular distribution of LC3 (Figure 5F). Consistently, the cells (both macrophages and malignant cells) in tumor grafts of LLC-bearing mice also exhibited elevated autophagy (Figure 6).

**FIGURE 7:**
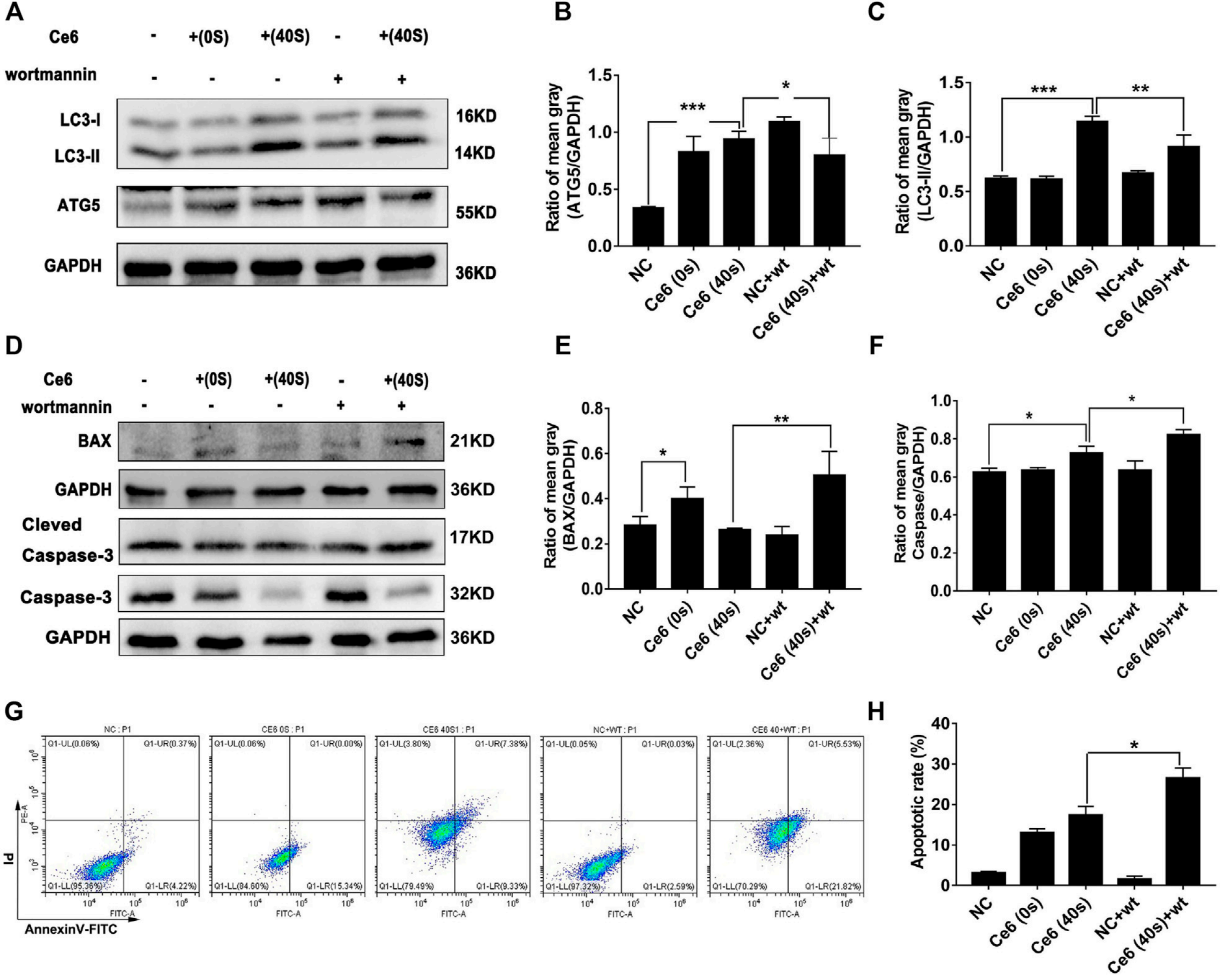
Autophagy inhibition resulted in the increased apoptosis of macrophages. Macrophages were pretreated with wortmannin (300 nM) for 1 h and then treated with Ce6, as described before. **(A–C)** The autophagy-related proteins (Atg-5, Beclin-1, and LC-3) were analyzed by WB. The blots were quantitatively analyzed using the ratio of mean gray. **(D–F)** The expression of Bax and caspase-3, which are biomarkers of apoptosis, was detected by WB. The blots were quantitatively analyzed using the ratio of mean gray. **(G,H)** The apoptosis rate (early apoptosis rate and late apoptosis rate) of macrophages was assayed by Annexin-V/PI staining and flow cytometry. The Annexin-V-positive cells were counted. Values were means ± SD (*n* = 3, *p < 0.05, **p < 0.01, ***p < 0.001, ****p < 0.0001).

Next, the inhibitor of autophagy, wortmannin, was applied to explore whether autophagy triggered by Ce6 PDT could protect macrophages from death. The results showed Ce6 PDT induced upregulation of Atg-5, and LC-3 II could be impaired by wortmannin (Figures 7A–C), an indication of effective autophagy inhibition. In terms of apoptosis, autophagy inhibition in Ce6-treated macrophages led to the enhanced expression of Bax and caspase-3 (Figures 7D–F). Further, the apoptosis rate of Ce6-treated macrophages increased as well (Figures 7G,H). In summary, PDT induced by Ce6 triggered autophagy of macrophages, which could effectively protect macrophages *per se* from death.

### Ce6-Mediated PDT Activated Molecules of the STING and Subsequent NF-κB

**FIGURE 8:**
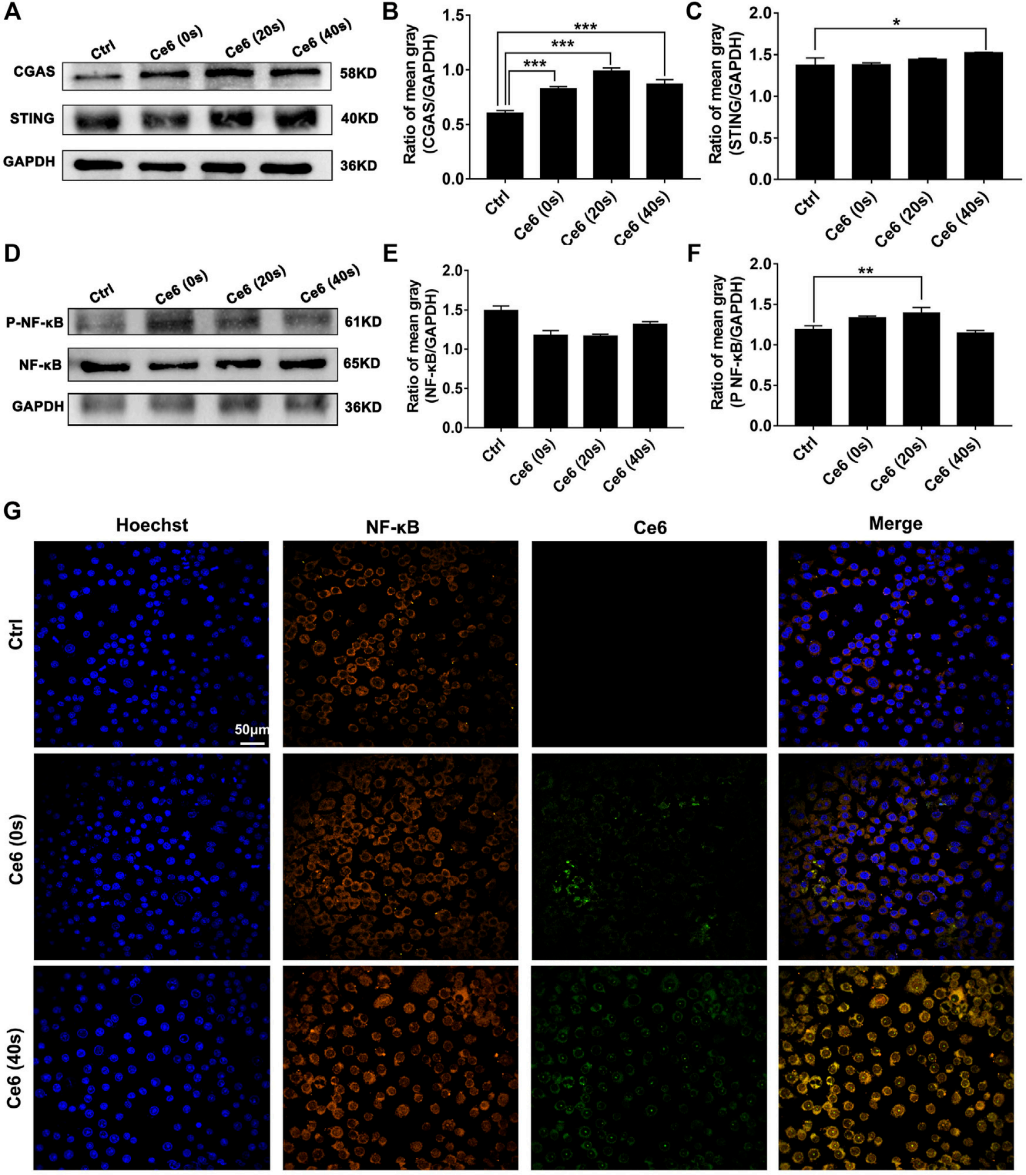
Photodynamic effect led to activation of the STING and NF-κB of macrophages *in vitro*. **(A–C)** Expression of cGAS and the STING in macrophages was detected by WB. The blots were quantitatively analyzed using the ratio of mean gray. **(D–F)** Expressions of NF-κB and p-NF-κB in macrophages were detected by WB. The blots were quantitatively analyzed using the ratio of mean gray. **(G)** NF-κB molecule in macrophages was labeled with immunofluorescence staining. Nuclear translocation of NF-κB was observed by confocal microscopy. Values were means ± SD (*n* = 3, *p < 0.05, **p < 0.01, ***p < 0.001, ****p < 0.0001).

**FIGURE 9:**
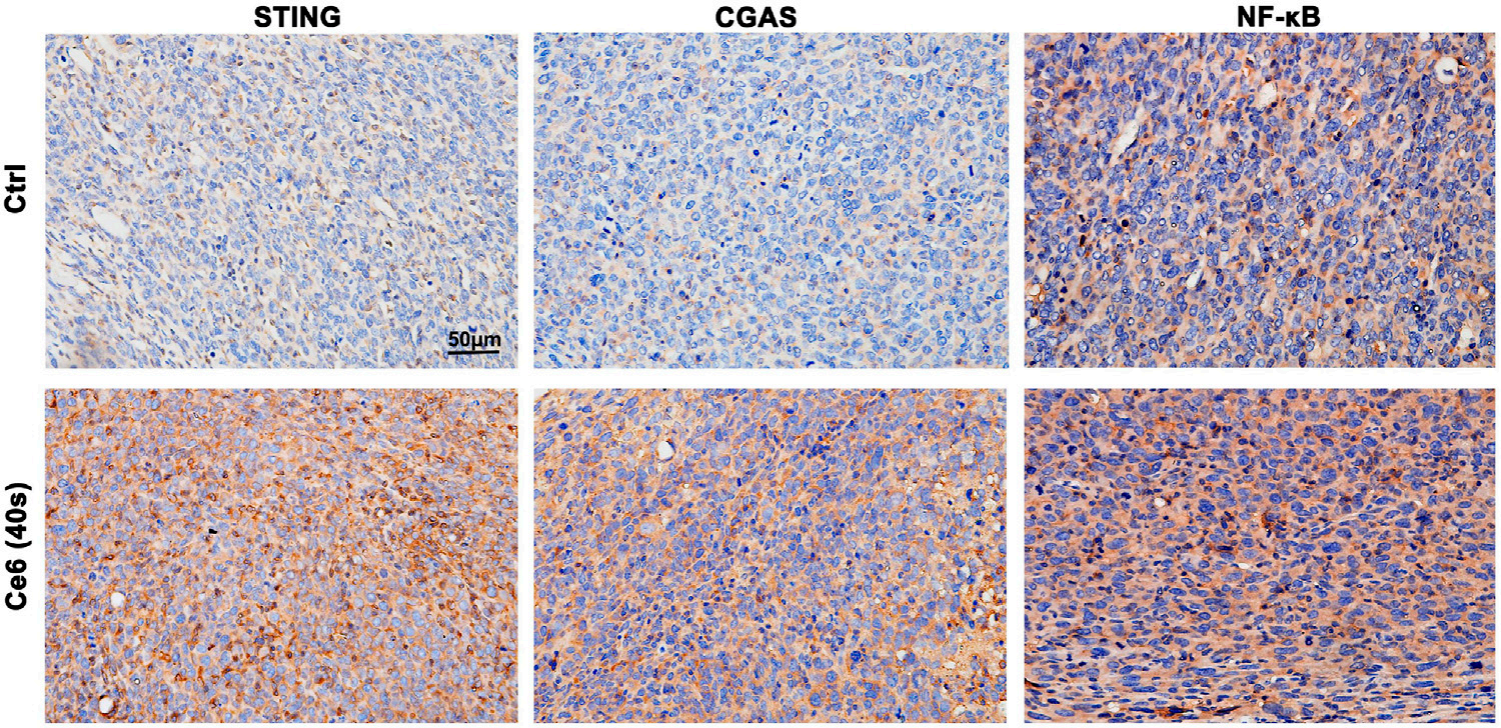
Photodynamic effect led to activation of the STING and NF-κB *in vivo*. STING, cGAS, and NF-κB expression in lung cancer tissues were analyzed by IHC staining. Ce6 PDT significantly activated cGAS/STING/NF-κB.

In addition to activating the autophagy signaling pathway, broken fragments from DNA damage also have the ability to activate the intracytoplasmic STING and subsequent NF-κB molecules (Chen et al., 2016; Li et al., 2018; Motwani et al., 2019; Kwon and Bakhoum, 2020). The activation of the STING and NF-κB in macrophages affected by Ce6 PDT was accessed thereupon. Not surprisingly, as shown in Figures 8A–F, the expression of cGAS, STING, and p-NF-κB was increased along with the prominent nuclear translocation of NF-κB in macrophages (Figure 8G), suggesting that molecules of the STING and NF-κB were activated. Consistent with the *in vitro* results, the evidence of IHC staining also showed strong activation of the STING and NF-κB in tumor grafts of LLC-bearing mice that received Ce6 PDT, implying that STING and NF-κB molecules were activated of cells (both macrophages and tumor cells) in the tumor grafts (Figure 9). In general, these results revealed PDT induced by Ce6 was a potent agent that could activate molecules of the STING and subsequent NF-κB in macrophages.

### Inhibition of ROS Attenuated Ce6 PDT-Mediated DNA Damage, STING Activation, and M1-Phenotype Remodeling of Macrophages

**FIGURE 10:**
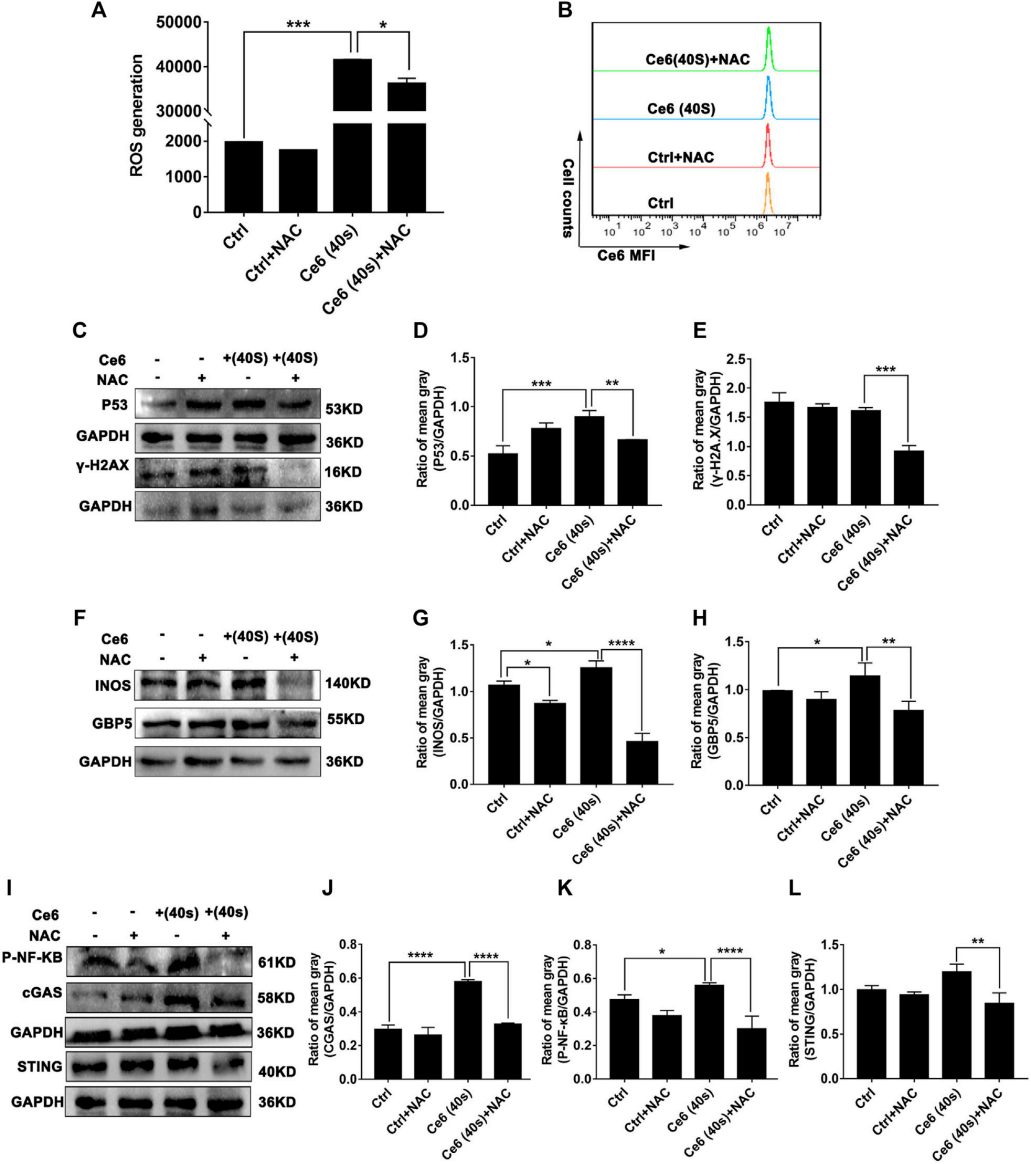
Blockage of ROS generation diminished Ce6 PDT-mediated DNA damage, STING activation, and M1-phenotype remodeling of macrophages. The ROS production was inhibited by NAC (3 mM) for 3 h and then treated with Ce6, as described in the previous parts. **(A,B)** ROS generation in macrophages was assayed by a DCFH-DA probe and flow cytometry. Geometric means were used to quantify the MFI. **(C–E)** The expression of DNA damage-associated proteins was determined by WB. The blots were quantitatively analyzed using the ratio of mean gray. **(F,H)** The expression of M1-phenotype biomarkers (GBP5 and iNOS) was detected by WB. The blots were quantitatively analyzed using the ratio of mean gray. **(I–L)** The expression of STING, cGAS, and p-NF-κB was analyzed by WB. The blots were quantitatively analyzed using the ratio of mean gray. Values were means ± SD (*n* = 3, *p < 0.05, **p < 0.01, ***p < 0.001, ****p < 0.0001).

The findings in the aforementioned section have strongly spoken that Ce6 PDT promoted ROS production in macrophages, damaged DNA, activated the STING, and reprogrammed macrophages into the M1 phenotype. To validate that Ce6 treatment-mediated oxidative damage is an essential driver of STING activation and reprogramming of macrophages to a pro-inflammatory phenotype, we next inhibited ROS in macrophages and then observed whether Ce6 treatment could still cause DNA damage, STING activation, and remodeling of the M1 phenotype. The results in Figures 10A,B showed that NAC pretreatment of macrophages was effective in reducing the intracellular ROS production. Additionally, NAC pretreatment simultaneously attenuated the DNA damage (Figures 10C–E) and STING activation caused by Ce6 treatment (Figures 10I–L). More importantly, M1 phenotype reprogramming induced by PDT in macrophages could also be antagonized by NAC (Figures 10F–H). In conclusion, blocking of Ce6-triggered ROS mitigated oxidative damage-driven STING activation and macrophage reprogramming to the M1 phenotype.

### Silence of the STING Molecule Impaired M1-Phenotype Reprogramming of Macrophages Induced by Ce6 PDT

**FIGURE 11:**
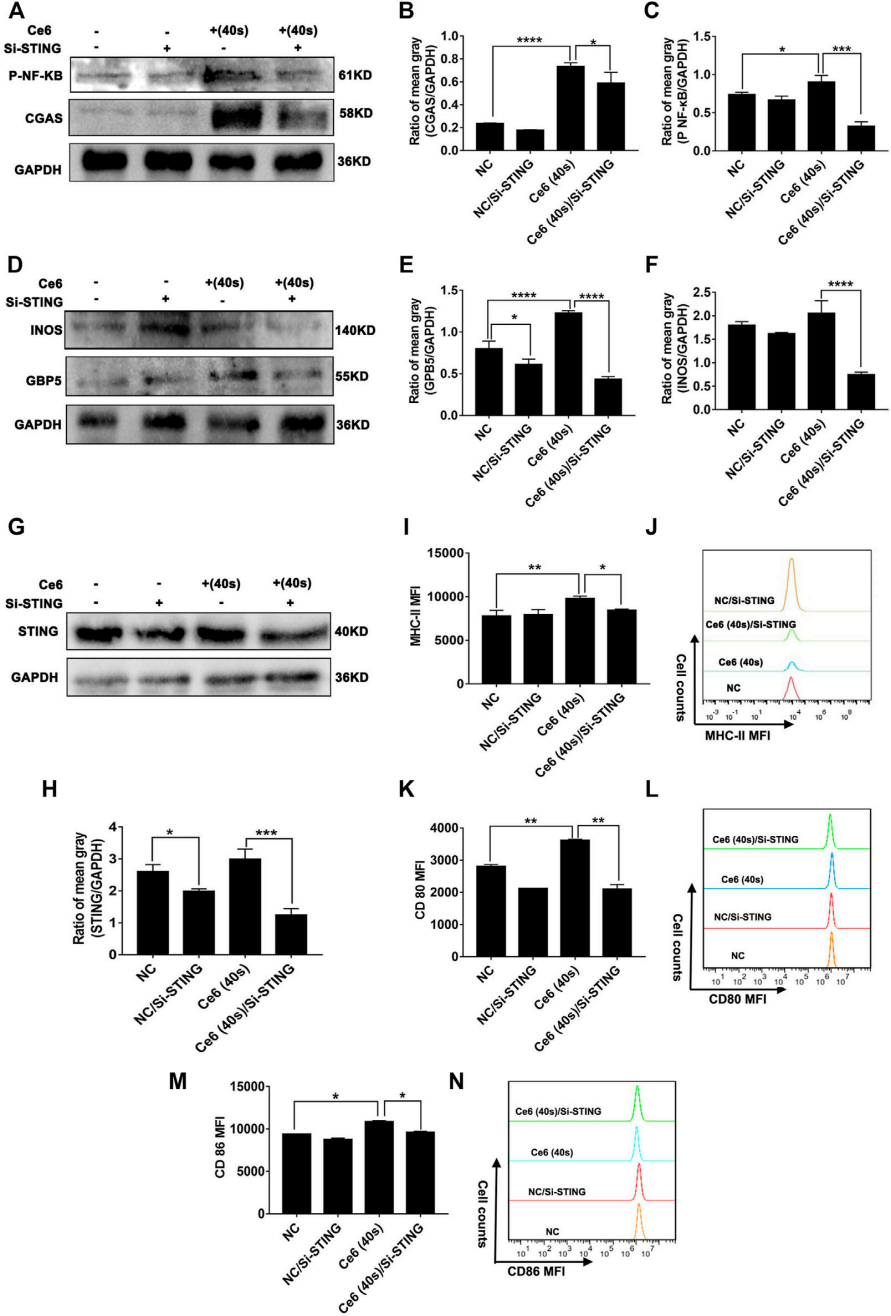
Suppression of the STING molecule attenuated the Ce6 PDT-induced M1 phenotype remodeling of macrophages. The STING molecule in macrophages was silenced by SiRNA (100 pM) for 8 h and then treated with Ce6, as described before. **(A–C)** The expression of cGAS and p-NF-κB in macrophages was measured by WB. The blots were quantitatively analyzed using the ratio of mean gray. **(D–F)** The molecules of iNOS and GBP5 were analyzed by WB. The blots were quantitatively analyzed using the ratio of mean gray. **(G,H)** The expression of the STING was detected by WB. The blots were quantitatively analyzed using the ratio of mean gray. **(I–N)** The surface expression of MHC-II, CD80, and CD86 was analyzed by immunofluorescence staining and flow cytometry. Geometric means were used to quantify the MFI. Values were means ± SD (*n* = 3, *p < 0.05, **p < 0.01, ***p < 0.001, ****p < 0.0001).

Apart from the driving factor of oxidative damage, the STING and its downstream NF-κB are key molecules that mediate the pro-inflammatory response of macrophages (Hou et al., 2018; Dunphy et al., 2018; Sun et al., 2020). The inflammatory response wherein macrophages is one of the essential contributors to their remodeling to the M1 phenotype (Lin et al., 2020). In light of Ce6 PDT’s effect on the STING and subsequent NF-κB, we proceeded to investigate the role of the STING molecule in the PDT-mediated reprogramming of macrophages to establish a logical link between them thereupon. The successful silencing of the STING can be verified by the downregulation of the STING and cGAS protein expression in Figures 11A,B,G,H. Based on this, as presented in Figures 11A,C, the silence of the STING was found to reduce the phosphorylation of NF-κB in Ce6-treated macrophages. More importantly, Ce6 PDT-induced M1-phenotype reprogramming of macrophages was abated by STING knockdown and characterized by the decreased expression of GBP5, iNOS, surface MHC-II, CD80, and CD86 (Figures 11D–F,I–N). Consequently, these findings indicated STING activation was probably a key driving factor in PDT-mediated phenotype remodeling of macrophages into the M1 phenotype after oxidative DNA damage.

## Discussion

**FIGURE 12:**
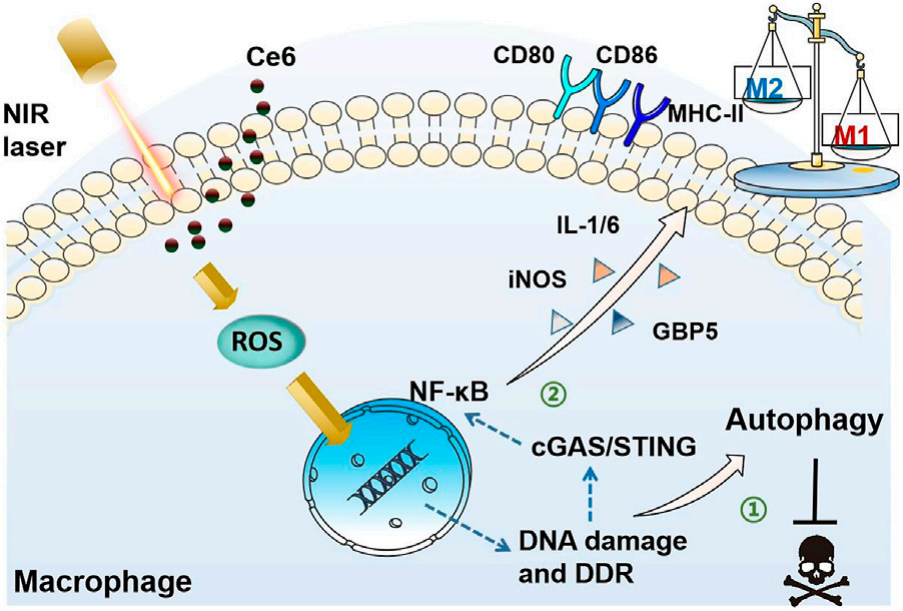
Schematic diagram of the mechanism of PDT induced by Ce6-mediated M1-phenotype reprogramming of macrophages. 1. PDT caused oxidative DNA damage and DDR triggered autophagy, thereof protecting macrophages from apoptosis. 2. Oxidative DNA damage activates cGAS/STING/NF-κB, leading to an enhanced pro-inflammatory phenotype.

In the present study, oxidative DNA damage was found to be triggered by the action of PDT on macrophages, which on the one hand activated autophagy and protected macrophages from death. On the other hand, DNA damage also activated the STING molecule, which serves as the crucial inflammatory response by activating its downstream NF-κB, thus polarizing macrophages into the M1 phenotype. Activation of STING and NF-κB molecules was critical for macrophage phenotypic reprogramming (Figure 12).

The photodynamic action initiated by the catalytic intracellular photosensitizer under certain conditions produces low concentrations of ROS and has little cytotoxicity. In the present study, we continued to use Ce6-mediated PDT to intervene in macrophages using the conditions from the previous study (Yu et al., 2021). Interestingly, low levels of ROS were generated and caused “low-intensity” damage to the DNA of the macrophages (Figures 3, 4). The intensity of the DNA damage determined whether the subsequent DNA damage response could repair damaged cells *per se*. High intracellular concentrations of ROS typically result in “high intensity” DNA damage, which is so severe that DDR is unable to repair the cell in time and apoptosis occurs thereupon (Galadari et al., 2017; Fendt and Lunt, 2020; Hanikoglu et al., 2020). In contrast, we used Ce6 to mediate the mild photodynamic action to generate low concentrations of ROS, which in turn caused “low intensity” DNA damage without cell death and induced effective DDR.

The downstream signaling of DDR is complicated, which includes the PI3K signaling pathway associated with autophagy (Galati et al., 2019). Our study revealed that Ce6-mediated PDT upregulated the autophagy level of macrophages (Figures 5, 6), whereas after inhibition of autophagy, Ce6-treated macrophages underwent apoptosis (Figure 7), suggesting that PDT stimulation of DDR-induced autophagy protected macrophages from apoptosis. However, we speculated that if the intensity of the photodynamic action is increased, the activation level of autophagy will be weaker than apoptosis; eventually, macrophage death will still occur.

Macrophages undergo autophagy in response to PDT treatment, maintaining their viability, which is a prerequisite for their phenotype to be remodeled. Another downstream molecule of DDR is the STING, a crucial molecule mediating the inflammatory response and the immune response to infection (Ma et al., 2020; Hopfner and Hornung, 2020; Reisländer et al., 2020). The STING molecule, known as a DNA sensor, can be activated by DNA from foreign viruses and by the tumor cells’ own DNA double-strand breaks and further promotes phosphorylation and nuclear translocation of NF-κB (Li and Chen, 2018). The activation of NF-κB promotes the inflammatory response of macrophages on the one hand, thereby effectively changing them from a pro-tumor M2 phenotype to a pro-inflammatory, anti-tumor M1 phenotype. NF-κB is also a nuclear transcription factor that boosts the expression of a series of proteins such as iNOS and GBP5 (Lin et al., 2020). These indicated that the STING molecule was centrally located in the PDT-mediated macrophage reprogramming process. As is well-known, inflammasome activation is a crucial pro-inflammatory pathway. In addition to the STING molecule, inflammasomes may be associated with the photodynamic action-mediated M1 polarization of macrophages. As far as we can speculate, NF-κB activation in macrophages, in addition to the mechanisms mentioned in the current study, can activate the NLRP3 inflammasome, which would activate caspase-1, prompting the conversion of pro-IL-1β to IL-1β and thereby reprogramming the macrophages. Additionally, the present study failed to use the method of co-staining macrophages (CD11b) with the relevant molecules (such as MHC-II or p53) in the histological detection of tumor grafts, which has limitations in determining whether the positively expressed proteins in the cancer tissues originated from macrophages or tumor cells.

## Conclusion

In summary, Ce6-mediated PDT stimulates macrophages undergoing oxidative DNA damage, which activates not only autophagy but also the STING molecule. Enhanced autophagy maintains viability of macrophages. Activation of the STING molecule promotes the inflammatory response of macrophages, leading to their reprogramming toward the M1 phenotype thereupon (Figure 12). The present work reveals the crucial mechanism by which the photodynamic action regulates the macrophage phenotype and also provides new intervenable signaling targets for remodeling macrophages into the M1 phenotype, which could be applied for anticancer immunotherapy in the future.

## Data Availability

The raw data supporting the conclusions of this article will be made available by the authors, without undue reservation.

## Supplementary Material

Supplementary Figure S1 Viability of macrophages at different laser irradiation time.

Supplementary Figure S2 Ce6 PDT upregulated the surface expression of CD80 and CD86 in mouse bone marrow-derived macrophages (mBMDMs).
