# Supplementary material for: Chlorin e6-Induced Photodynamic Effect Polarizes the Macrophage Into an M1 Phenotype Through Oxidative DNA Damage and Activation of STING
Source: Front Pharmacol. 2022 Mar 3;13:837784. doi: 10.3389/fphar.2022.837784 (PMC8927874; doi:10.3389/fphar.2022.837784)
Supplement: Supplementary file 1 [file DataSheet1.docx]

**Supplementary Material**

**
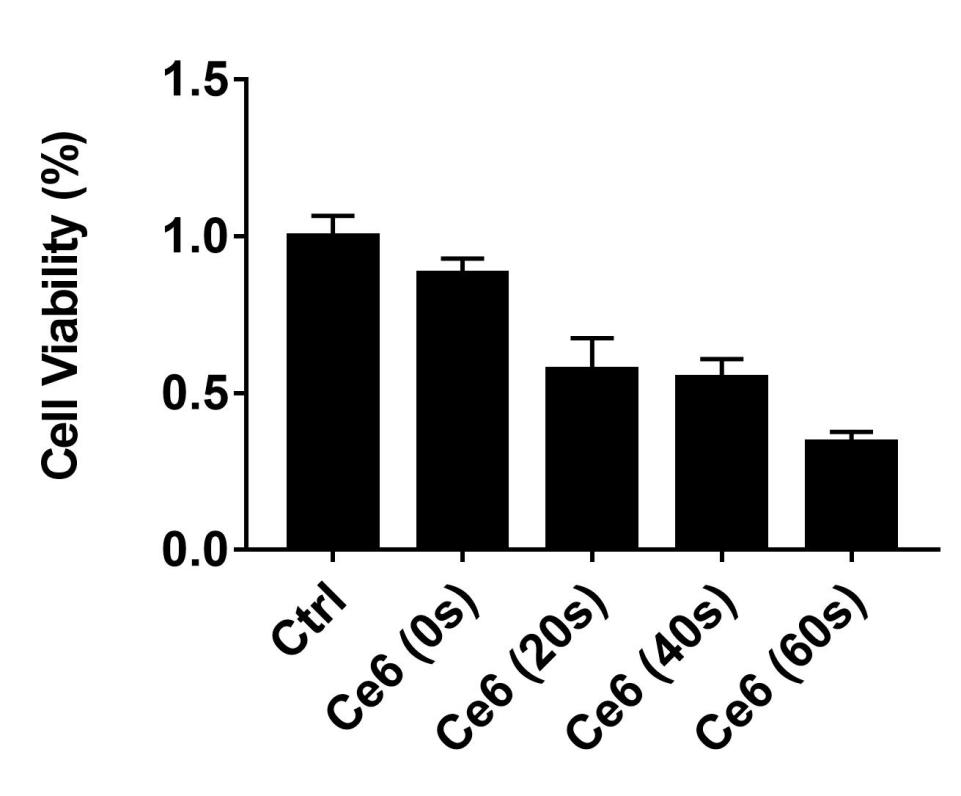
**

**Fig. S1. Viability of macrophages at different laser irradiation time.**

**
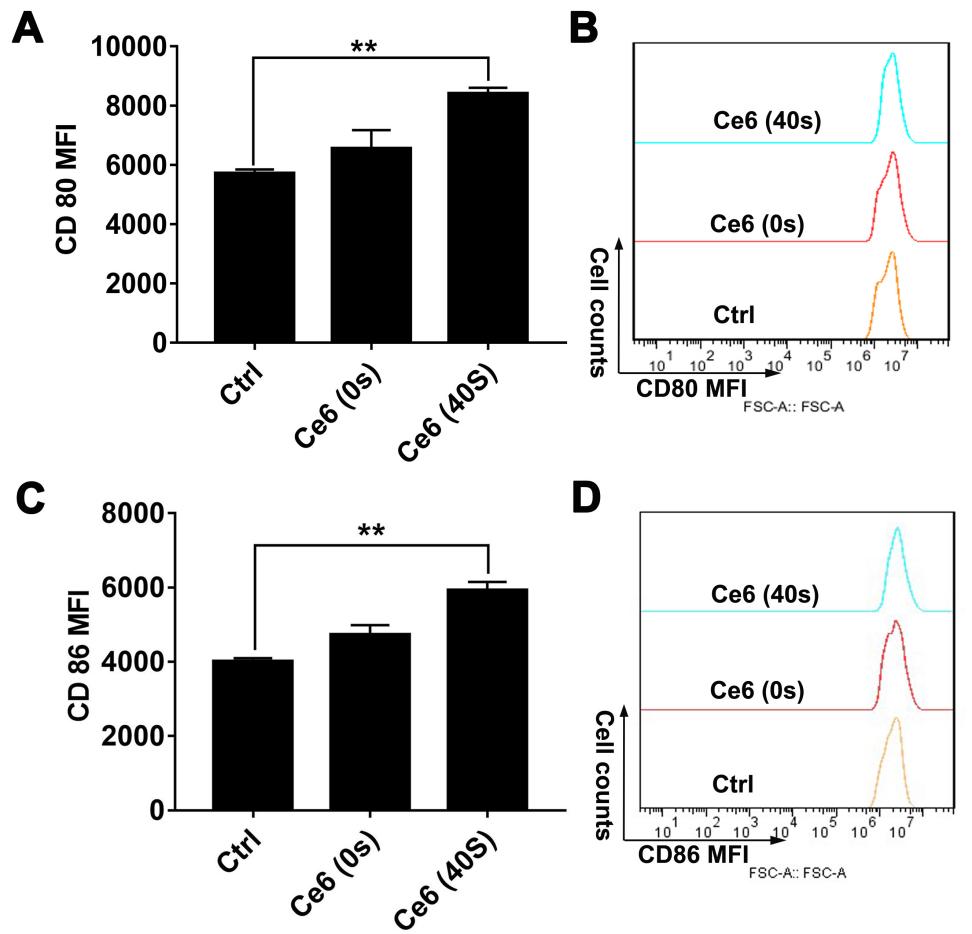
**

**Fig. S2. Ce6 PDT up-regulated the surface expression of CD80 and CD86 in mouse bone marrow derived macrophages (mBMDMs).**
